# Around the Hospitals

**Published:** 1895-01-12

**Authors:** 


### AROUND THE HOSPITALS.

[NOTICE TO THE MANAGERS OF INSTITUTIONS.—Special space is now reserved for the insertion of notices of hospital meetings and festivals. The Editor requests that all notices may reach THE HOSPITAL Office, 428, Strand, at least one week before the date of each meeting.]

**The Doncaster Infirmary and Dispensary.**—Work appears to continue without very marked changes at the infirmary. It is true that in the out-patient department a certain increase is visible, but this does not apply to the sick within the wards, where 153 patients were admitted. We hope this is an indication that sickness was not rife in Doncaster in 1893. The institution receives fair support from the neighbourhood, but the collieries do not seem to have co-operated to any appreciable extent as yet, though they have come in largely for the benefits of the hospital. Various societies and friends have been particularly active, and the subscribers' list is a considerable one. Amongst legacies, those of Mrs. Stockill, £400, and Mrs. Till £350, are the most considerable. Referring to legacies as a source of revenue, Mr. Brundell remarked at the annual meeting of the institution, that they should be added to investments for the endowment of the hospital. This is laying down a hard and fast rule, which is open to dispute. As years go on, and legacies accumulate, future generations would under this system be absolved from the necessity of providing for the sick of their generation. If a sufficient sum were set aside to defray a reasonable and definite proportion of the yearly expenditure, or to provide for bad seasons, the reserve funds might well be appropriated to extension and improvements, so often now necessitating special appeal. The Doncaster Infirmary expends £1,700 yearly, and of this sum they have a secured income of over £400. The difference should certainly be forthcoming from the neighbourhood, and if efforts are not relaxed, generous contributions will continue to maintain its present condition. The institution is especially fortunate in having secured the assistance of energetic honorary secretaries. It is no easy matter to obtain voluntary service of real value.

**The Royal Portsmouth, Portsea, and Gosport Hospital.**—Everywhere throughout the report of this hospital a desire to secure careful administration is visible. This is shown in one way by a comparison of the expenditure tables for the two past years. Whilst the patients numbered 123 above those admitted in the previous year, the expenditure was diminished by over £300. The out-patients too were more than 800 above the number last recorded, and, therefore, whilst in most cases the various items of expenditure were reduced, drugs and dressings show an increase. The Hospital Sunday and Saturday Funds give very substantial support to the institution, and last year the Duke of Connaught was a generous donor on his retirement from the command of the district. Further, the Mayoress held a ball on behalf of the hospital, which added £100 to its funds. The patients numbered 1,088, whilst out-patients of all descriptions amounted to 8,896. The ordinary income was £5,851, the expenditure £5,633. The cost per bed occupied was £43 9s. 7d., a result which places the Portsmouth Hospital in the foremost ranks of economy. We notice that the hospital has not adopted the uniform system of accounts, a fact to be regretted, as a desire to give full and accurate detail is apparent, and uniformity would more strongly bring out a comparison in favour of the institution.

